# Examining Depressive Symptoms and Their Predictors in Malaysia: Stress, Locus of Control, and Occupation

**DOI:** 10.3389/fpsyg.2017.01411

**Published:** 2017-08-22

**Authors:** Si H. Yeoh, Cai L. Tam, Chee P. Wong, Gregory Bonn

**Affiliations:** ^1^Department of Psychology, Jeffrey Cheah School of Medicine and Health Sciences, Monash University Malaysia Bandar Sunway, Malaysia; ^2^Psychology, Department of General Studies, King Fahd University of Petroleum and Minerals Dhahran, Saudi Arabia

**Keywords:** depression, stress, locus of control, occupation, Malaysia

## Abstract

The 2015 National Health and Morbidity Survey estimated that over 29% of the adult population of Malaysia suffers from mental distress, a nearly 3-fold increase from the 10.7% estimated by the NHMS in 1996 pointing to the potential beginnings of a public health crisis. This study aimed to better understand this trend by assessing depressive symptoms and their correlates in a cross-section of Malaysians. Specifically, it assesses stress, perceived locus of control, and various socio-demographic variables as possible predictors of depressive symptoms in the Malaysian context. A total of 728 adults from three Malaysian states (Selangor, Penang, Terengganu) completed Beck’s depression inventory as well as several other measures: 10% of respondents reported experiencing severe levels of depressive symptoms, 11% reported moderate and 15% reported mild depressive symptoms indicating that Malaysians are experiencing high levels of emotional distress. When controlling for the influence of other variables, depressive symptoms were predictably related to higher levels of stress and lower levels of internal locus of control. Ethnic Chinese Malaysians, housewives and those engaged in professional-type occupations reported less depressive symptoms. Business owners reported more depressive symptoms. Further research should look more into Malaysians’ subjective experience of stress and depression as well as explore environmental factors that may be contributing to mental health issues. It is argued that future policies can be designed to better balance individual mental health needs with economic growth.

## Introduction

The most recent Malaysian National Health and Morbidity Survey (Institute for Public Health, 2015) estimated that 29.9% of adults in Malaysia are experiencing mental health problems such as depression and anxiety. This number represents an alarming increase from the prevalence of 10.7% estimated by the NHMS in 1996. If accurate, such a dramatic increase in mental health problems demands further scrutiny. To this end, the current study looked at the prevalence of depressive symptoms in several of the most populous areas of Malaysia.

Depression, the most common form of mental illness, relates to impaired overall functioning, decreased quality of life, and a variety of health problems (Rapaport et al., 2005; Lim et al., 2012; Johansson et al., 2013). As a cause of morbidity, depression consistently ranks as one of the costliest diseases, accounting for approximately 12% of years lived with disability worldwide (World Health Organization [WHO], 2008), and it is expected to be the most common debilitative disorder in high income countries by the year 2030 (World Health Organization [WHO], 2001). Mood disorders, including depression, are also a leading cause of suicide, accounting for 35.8% worldwide (Bertolote and Fleischmann, 2002).

Given the well-documented health problems, physical distress, functional impairment, suicide risks, and burden to caregivers that surround depression and other mental health problems (Maynard, 2003; Moussavi et al., 2007; Strine et al., 2009), the recent NHMS figures could point to the beginnings of a serious public health crisis. The current study analyzed data collected for a larger project (see, for example, Tam et al., 2016). The primary aim was to provide a more accurate estimate of the prevalence of depressive symptoms in particular (as opposed to mental health problems in general) by looking at results from Beck’s Depression Inventory (BDI-II; Beck et al., 1996). Beyond this, the study used the existing data set to gain some indication as to which subgroups within Malaysian society are most at risk for depression. Because the selection of variables was limited by those included in a larger survey, the analyses presented here are not meant to be an exhaustive or ideal representation of the many correlates of depression. Rather, they are meant as a starting point for understanding the prevalence of depression among different groups of Malaysians and thus as a potential guide for future research. The variables included and related findings from previous studies are discussed in the following section.

## Background

### Depression

Depression is defined as the presence of depressed mood and/or the loss of interest/pleasure, combined with five or more of the following symptoms: changes in appetite or weight, sleeping problems, psychomotor agitation or retardation, fatigue, feelings of worthlessness, loss of concentration, or recurrent suicidal thoughts. For a diagnosis of major depressive order to take place, these symptoms should represent a change from previous functioning and last for a period of at least 2 weeks (American Psychiatric Association [APA], 2013). In the United States, the 12-month prevalence of major depressive disorder has been estimated to be around 7% with significant variation by age and ethnicity. For example, among 18–29-year-olds depression is three-times as common as it is among those 60 years or older, and Asian Americans report much lower incidence of severe depressive symptoms (around 4%) compared to Whites and Native Americans (8–9%) (American Psychiatric Association [APA], 2013).

By contrast, a brief review of studies conducted in Malaysia showed generally higher rates of severe depressive symptoms (i.e., those suggestive of a major depressive disorder). These findings, however, are somewhat difficult to interpret because the studies conducted in Malaysia use numerous different measures of depression as well as inconsistent samples. For example, one study estimated an 11.3% rate of depression using the Hospital Anxiety and Depression Scale (Wong and Lua, 2011). Another study using the Patient Health Questionnaire-9 reported a 12.3% rate of depression among urban poor in Peninsular Malaysia (Tan and Yadav, 2015), whereas a different study using the same scale estimated a depression rate of 10.3% among a general sample of adults in the state of Selangor (Kader Maideen et al., 2014). A recent study in the state of Penang (using the Geriatric Depression Scale) found that 19.2% of its elderly participants were experiencing severe depressive symptoms (Rashid and Tahir, 2015). Thus, all of these studies do appear to indicate that depressive symptoms are more common in Malaysia than in the United States. It is not clear, however, if the prevalence rate in Malaysia is closer to 11 or 19%. It is also not clear from these studies why people in Malaysia are experiencing depression. One possible explanation for the inconsistent findings is that these previous studies often used measures of general well-being rather than those specifically designed to assess depressive symptoms. The current study aimed to address these issues by using Beck’s Depression Inventory (BDI-II; Beck et al., 1996) and a larger, more diverse sample of otherwise healthy adults. It also looked into potential causes of depression in Malaysia by looking at some common correlates which are discussed below.

### Psychological Factors

#### Stress

Research shows that stress is an important contributor to depression (Richards et al., 2016). Although stress is the body’s natural response to any kind of demand or threat, when experienced at high levels, or over extended periods of time, it negatively affects mood, causes irritability, disrupts sleep patterns, and interferes with concentration. Research in Malaysia has shown that stressors such as problems at work, unhappy family relationships, and financial problems relate to depression (Rusli et al., 2008; Kader Maideen et al., 2014). Other research has shown that high levels of perceived stress predict more severe depressive symptoms (Bergdahl and Bergdahl, 2002; Sangon, 2004).

#### Health Locus of Control (HLOC)

Locus of control is the degree to which people believe that they have control over events in their lives and it has often been hypothesized as a contributory factor in depression (Rotter, 1966). People with internal locus of control tend to attribute events and outcomes to their own actions while those with external locus of control blame outside forces such as chance, destiny, or more powerful individuals. Levenson (1974) proposed three independent dimensions to locus of control: Internality (internal control) refers to the believe that life’s outcomes are determined by one’s own behaviors. Powerful others (external control) refers to the belief that outcomes are controlled by other people. Chance, finally, refers to the belief that outcomes are largely random. Wallston et al. (1978) created the Multidimensional Health Locus of Control Scale as a means of measuring beliefs about each of these three dimensions. Research using this measure has demonstrated that people with an internal locus of control (internals) take more personal responsibility, explore more effective coping techniques and are able to delay gratification better than people with external locus of control (externals). Lefcourt (1976) thus argued that internals are less prone to mental illness. Externals, on the other hand, tend to believe that their actions have little effect on their situation. Therefore, they tend more toward feelings of helplessness, depression and anxiety than internals.

Research supports the proposition that locus of control relates to depression. Aflakseir and Mohammad-Abadi (2016), for example, found that, among 108 retirees, depression related to lower internal HLOC, although powerful-others and chance HLOC did not specifically predict depression. Another study, involving 10,302 respondents from the Netherlands, found that increases in external locus of control predicted more depressive symptomology while increases in internal locus of control predicted lower levels of depressive symptoms (van Dijk et al., 2013). Overall, a meta-analysis of 40 years’ worth of research on locus of control shows a moderately strong positive relationship between external LOC and depressive symptoms (Cheng et al., 2013). Important for the purposes of this study, the same meta-analysis also indicated that LOC may operate somewhat differently for Asians compared to Westerners: In some Asian cultures a positive relationship seems to exist between fate/chance control and external control. Also, although many Asians believe that outcomes are ultimately determined by fate they still feel that it is important to strive for better outcomes.

### Socio-Demographic Factors

Socio-demographic differences in depression have been evident in previous studies conducted in Malaysia (Kader Maideen et al., 2014; Kaur et al., 2014; Rashid and Tahir, 2015; Tan and Yadav, 2015). However, findings from these studies are difficult to interpret given that the various samples were limited in terms of age group and geographical areas. Part of the rationale for this study, thus, was to investigate how various socio-demographic factors relate to depressive symptoms among a larger, more inclusive sample of Malaysians.

#### Age

A study of Malaysians aged 60–69 years among 2005 participants revealed that the prevalence of depression increased with age (Rashid and Tahir, 2015). Most studies, however, indicate that depression is more common among younger age groups (Tan and Yadav, 2015). For example, American 18-to-29-year-olds are three times more likely to be depressed than adults aged 60 and above (American Psychiatric Association [APA], 2013).

#### Gender

NHMS results (2015) indicate that mental health problems are more common among Malaysian females (30.8%) than males (27.6%). Other samples of Malaysians, however, have produced different results: Malaysian males from a sample of the urban poor were 1.5 times more likely than females to be depressed (Tan and Yadav, 2015) while other studies have found no gender differences (Wong and Lua, 2011). Again, differences in sampling and the types of measures used are probably contributing to these inconsistencies.

#### Ethnicity

The population of Malaysia is quite diverse, but it is normally thought of as containing three major ethnic groups: About 58% of the population is Malay, with Chinese and Indian ethnicities accounting for another 27 and 8% respectively. The remaining 10% is made up of various other groups. Varying rates of depression among these different ethnic groups have been reported. Again, however, results have not been consistent from study to study. Kader Maideen et al. (2014), for example, reported the highest prevalence of depression among those who self-identified as “other” in terms of ethnicity (17.6%), followed by those who identified as Chinese (13.8%), Malay (10.8%), and Indian (6.1%). Yusoff et al. (2014), on the other hand, found those of Indian descent to be most depressed, followed by ethnic Chinese, as did Kaur et al. (2014) in his study of those aged 60–69. Another study looking at Malaysian university students found no ethnic differences (Fuad et al., 2015).

#### Marital Status

Generally, marriage is found to be protective against depression. This appears to be true in Malaysia as well. For example, a survey of 1556 Malaysian adults revealed that depression was more prevalent among divorcees (42.9%) compared to those who were separated (33.3%), singles (14.0%), and widowed (11.5%). Married couples had the lowest rate at 7.8% (Kader Maideen et al., 2014). Earlier studies of elderly samples in Pakistan, Malaysia, and Brazil also reported increased risk of depression among those who were not married (Djernes, 2006; Blay et al., 2007; Taqui et al., 2007; Imran et al., 2009). One study of the elderly in Malaysia found, however, that married participants had a higher prevalence of severe depression, possibly due to the demands of caring for their aging spouses (Rashid and Tahir, 2015).

#### Education, and Occupation

Few studies have looked at the relationships of educational level and occupation to depression in Malaysia. However, Rashid and Tahir’s (2015) study of the elderly found that severe depression was highest among those with just primary school education or less. The same study also found that elderly Malaysians who were employed were less likely to experience severe depression.

## Objectives

The objectives of this study were: (1) To gain a better understanding of the prevalence of depressive symptoms among Malaysians in general. (2) To provide some insight into the roots and correlates of depression among Malaysians

## Research Questions

(1) What is the prevalence of depressive symptoms among Malaysian adults?
(2) How do stress and HLOC relate to depressive symptoms?
(3) What sociodemographic factors (i.e., age, gender, ethnicity, education, marital status, and occupation) are predictive of depressive symptoms in Malaysia?

## Materials and Methods

### Study Design

This was a cross-sectional study using a self-report survey questionnaire. Ethics approval was obtained from the Monash University Human Research Ethics Committee (MUHREC: CF12/3382-2012001623). All participants were over 18 and signed a standard informed consent agreement before completing the survey.

### Participants

All participants in this study were residents of Malaysia over 18 years of age. A total of 794 people completed the questionnaire. Of these, 66 surveys were excluded from analysis due to incomplete responses, severe medical conditions, or non-compliance with instructions. Final analyses, thus, included 728 participants from three Malaysian states – Penang, Selangor, and Terengganu. Ages ranged from 18 to 77 years old (*M* = 30.76, *SD* = 13.22). There were 273 males and 455 females. All participants were Malaysian residents; 36% were ethnic Malays, 42.4% were ethnic Chinese, 18.1% were ethnic Indians, and 2.1% were of other, or undefined, ethnicity (refer **Table 1**). Other socio-demographic details are presented in **Table 1**.

**Table 1:** Descriptive statistics of participants.

Variable	Frequency (Percentage)	Mean age	Mean of depression score (*SD*)
**Gender**			
Male	273 (37.5%)	31.38	12.08 (11.66)
Female	455 (62.5%)	28.39	11.60 (10.29)
**Race**			
Malay	262 (36.0%)	27.04	12.35 (11.05)
Chinese	309 (42.4%)	28.29	10.79 (10.08)
Indian	132 (18.1%)	37.34	12.80 (11.98)
Others	15 (2.1%)	26.59	11.87 (8.32)
**Marital status**			
Single	463 (63.6%)	24.07	12.21 (10.88)
Married	234 (32.1%)	42.00	10.64 (10.48)
Divorced	14 (1.9%)	41.41	17.79 (12.79)
Widowed	13 (1.8%)	42.38	11.23 (10.99)
Others	2 (0.3%)	21.00	7.00 (8.49)
**Education**			
Primary school	33 (4.5%)	45.64	14.70 (13.48)
High school	368 (50.5%)	32.17	11.56 (10.99)
Pre-university	134 (18.4%)	28.06	11.99 (11.10)
University degree and above	184 (25.3%)	25.39	11.58 (9.82)
Others	4 (0.5%)	52.50	8.50 (8.89)
**Occupation**			
Housewife	50 (6.9%)	47.74	8.92 (9.63)
Student	184 (25.3%)	20.95	12.29 (8.99)
Business owner	53 (7.3%)	42.30	15.04 (12.66)
Professional	44 (6.0%)	33.44	6.18 (8.14)
Administrator	20 (2.7%)	34.43	13.60 (13.72)
Sales/service	261 (35.9%)	29.17	12.19 (11.74)
Others	106 (14.6%)	36.62	11.80 (10.45)

### Measures

#### Beck Depression Inventory–II (BDI-II; Beck et al., 1996)

The BDI-II was used to assess participants’ level of depressive symptoms. The BDI-II consists of 21 items, and uses a 4-point Likert scale scored from 0 to 3. Questions assess the severity of various depressive symptoms such as sadness, pessimism, past failure, loss of pleasure, etc. All scores are summed into a total score. A higher total score indicates more severe depressive symptoms. Four levels of symptom severity can be derived from the total score – minimal, mild, moderate, and severe. The BDI-II reported a coefficient alpha rating of 0.92 for outpatients and 0.93 for college student samples (Beck et al., 1996) This inventory correlates positively with the Hamilton Depressing Rating Scale, *r* = 0.71, and has a 1-week test–retest reliability of *r* = 0.93 and an overall internal consistency of α = 0.91 (Beck et al., 1996).

#### Simple Lifestyle Indicator Questionnaire (SLIQ; Godwin et al., 2008)

Simple Lifestyle Indicator Questionnaire is a 12-item instrument, which assesses five domains: Diet, physical activity, alcohol consumption, stress, and smoking. Stress level is assessed via a six-point Likert scale ranging from 1 to 6 with higher scores indicating higher stress levels. The test–retest reliability for the 12 questions ranged, in previous studies, from 0.63 to 0.97 (Godwin et al., 2008). A correlation coefficient of 0.77 between participants’ and blinded rater’s scores on the SLIQ was reported (Godwin et al., 2008).

#### Multidimensional Health Locus of Control Scale (MHLC; Wallston et al., 1978)

The MHLC was used to assess locus of control, or perceived level of personal control, over health-related behavior. It is an 18-item instrument that measures three dimensions of locus of control: Internality of health locus of control; powerful-other HLOC; and chance HLOC. All 18 items are arranged on a 6-point Likert scale ranging from “strongly agree” to “strongly disagree.” Higher scores for a dimension indicate a stronger belief in that dimension of HLOC. In previous studies the internal reliability (Cronbach’s α) for this scale ranges from 0.67 to 0.77 for all three dimensions. This scale also has good criterion validity, correlating with participants’ state of health. Heath related behavior such as exercise was highly associated with “internal” health belief whereas adverse health related behaviors such as smoking and drinking were associated with “external” health belief (Kuwahara et al., 2004).

#### Demographic Information

Participants reported their gender, age, ethnicity, and educational level. No personally identifiable information (e.g., name, IC number) was collected to ensure the confidentiality of the participants and their responses. Participants were given contact information for the researchers if they had questions or wished to be informed of the research results.

### Procedure

Participants were recruited at public places such as shopping areas and malls from three different states: Penang, Selangor, and Terengganu. Prior to answering the questionnaire, all participants read a statement of purpose and provided their consent. Participants were paid 10 ringgit Malaysia (about 2.5 USD) for their participation. The survey took most people 20–30 min to complete.

### Statistical Analyses

Descriptive analysis was used to assess overall levels of depressive symptoms, locus of control, and life stress. Pearson’s bivariate correlation was used to examine relationships between depression and demographic factors with continuous data (i.e., age, education level), as well as psychological factors (i.e., stress and HLOC – internal, change, and powerful-others). *T*-tests were conducted to examine gender differences in depression, and point-biserial correlations were used to examine the relationships between depression and demographic factors with categorical data (i.e., ethnicity, occupation, and marital status). Lastly, multiple linear regressions were conducted to examine the unique effects of intercorrelated factors on depression.

## Results

### Descriptive Data

Descriptive analysis was conducted to examine Malaysians’ level of depressive symptoms, HLOC, and stress level (refer **Table 2**).

**Table 2:** Descriptive data of measurements (*N* = 728).

Variable	Score range	Mean (*SD*)	Frequency (%)
**Depression**			
Minimal	0 – 13		465 (63.9%)
Mild	14 – 19		110 (15.1%)
Moderate	20 – 28		80 (11.0%)
Severe	29 – 63		73 (10.0%)
**Health locus of control**			
Internal	1 – 6	4.51 (0.92)	
Chance	1 – 6	3.42 (0.97)	
Powerful-others	1 – 6	3.80 (1.00)	
Life stress score	1 – 6	3.33 (1.29)	

#### Depression Severity by Ethnicity

Levels of depressive symptoms among the different ethnic groups are presented in **Table 3** below. Overall depression rate, calculated by summing the percentage of moderate and severe depression categories for each ethnicity, was as follows: 10.3 and 11.1% of ethnic Malays had severe and moderate depression respectively, which translates to an overall rate of depression of 21.4% for Malays. 7.4 and 11.7% of ethnic Chinese participants reported having severe and moderate depression respectively. Thus the overall depression rate among ethnic Chinese participants was 19.1%. A depression rate of 23.5% was found for ethnic Indians of whom 15.2% reported severe, and 8.3% reported moderate depressive symptoms. Those in the “Other” ethnic category had the highest overall depression rate (26.7%) with 6.7% reporting severe depressive symptoms and 20.0% reporting moderate depressive symptoms.

**Table 3:** Descriptive data of depression severity by ethnicity (*N* = 718).

Depression severity	Ethnicity				Total
	Malay	Chinese	Indian	Others	
Minimal	163 (62.2%)	205 (66.3%)	82 (62.1%)	10 (66.7%)	460 (64.1%)
Mild	43 (16.4%)	45 (14.6%)	19 (14.4%)	1 (6.7%)	108 (15.0%)
Moderate	29 (11.1%)	36 (11.7%)	11 (8.3%)	3 (20.0%)	79 (11.0%)
Severe	27 (10.3%)	23 (7.4%)	20 (15.2%)	1 (6.7%)	71 (9.9%)
Total	262 (100.0%)	309 (100.0%)	132 (100.0%)	15 (100.0%)	718 (100.0%)

### Relationship between Depression, Age, Education Level, Stress, and HLOC

Pearson’s bivariate correlation was used to examine the relationships among depression, age, education level, stress, and HLOC (i.e., internal, chance, and powerful-others). Results showed a significant positive relationship between depression and stress, *r* = 0.177 (*N* = 718), *p* < 0.01, indicating that people with higher stress were more depressed. Significant negative relationships were also shown between depression and age, *r* = -0.092 (*N* = 727), *p* < 0.05, internal HLOC, *r* = -0.188 (*N* = 728), *p* < 0.01, and powerful-others HLOC, *r* = -0.142 (*N* = 728), *p* < 0.01. In other words, people who were older, or had higher internal or powerful-others HLOC were less depressed. No significant relationships were found between depression and education level, *r* = -0.021 (*N* = 718), *p* > 0.05, or chance HLOC, *r* = -0.019 (*N* = 728), *p* > 0.05 (see **Table 4**).

**Table 4:** Correlation between Depression, Age, Education Level, Stress, and HLOC (Internal, Chance, and Powerful-Others)

Variable	Age	Education Level	Stress	Internal HLOC	Chance HLOC	Powerful-Others HLOC
Depression	-0.092^∗^	-0.021	0.177**	-0.188**	-0.019	-0.142**
Age		-0.278*	-0.112**	0.081*	0.045	0.172**
Education Level			0.214**	0.182**	-0.076*	-0.137**
Stress				0.029	-0.076*	-0.123**
Internal HLOC					0.324**	0.405**
Chance HLOC						0.539**

^∗^*p < 0.05, ^∗∗^*p <* 0.01*.

### Gender Differences in Depression

A *t*-test was conducted to examine gender differences in depression. Results showed no significant difference between males (*M* = 12.08) and females (*M* = 11.60) in depressive symptoms, *t*(726) = 0.577, *p >* 0.05 (see **Table 5**).

**Table 5:** Independent *t*-test comparing gender differences in depression.

Variable	Mean scores		
	Male	Female	*t*(N)
Depression	12.08	11.60	0.577 (726)

### Relationship between Depression and Ethnicity

Point-biserial correlations were conducted to examine the relationship between depression and ethnicity There was a significant negative relationship between depression and Chinese, *r_pb_* = -0.077 (*N* = 718), *p* < 0.05, with a *R*^2^ of 0.006. This indicates that Chinese were less depressed compared to other ethnicities. No significant relationship was found between depression and Malay, *r_pb_* = 0.042 (*N* = 718), *p* > 0.05 and Indian, *r_pb_* = 0.046 (*N* = 718), *p* > 0.05 (see **Table 6**).

**Table 6:** Point-biserial correlation between depression and ethnicity, marital status, and occupation.

Variable	Depression (*r_pb_*)
**Ethnicity**	
Malay	0.042
Chinese	-0.77*
Indian	0.046
**Occupation**	
Housewife	-0.073*
Student	0.026
Business owner	0.084*
Professional	-0.133**
Admin	0.028
Marital status	0.027
Single	0.053
Married	-0.073*
Divorced	0.078
Widowed	-0.007

^∗^*p < 0.05, ^∗∗^*p <* 0.01*.

### Relationship between Depression and Occupation

Point-biserial correlations were conducted to examine the relationship between depression and occupation status. There was a significant positive relationship between depression and business owners, *r_pb_* = -0.084 (*N* = 718), *p* < 0.01, with a *R*^2^ of 0.007, whereas significant negative relationships were shown between depression and housewives, *r_pb_* = -0.073 (*N* = 718), *p* < 0.05, with a *R*^2^ of 0.005, and professionals, *r_pb_* = -0.133 (*N* = 718), *p* < 0.01, with a *R*^2^ of 0.018 (see **Table 6**). These results indicated that compared to other occupations, housewives and professionals were less depressed whereas business owners were more depressed.

### Relationship between Depression and Marital Status

Point-biserial correlations were conducted to examine the relationship between depression and marital status. There was a significant positive relationship between depression and being divorced, *r_pb_* = 0.078 (*N* = 726), *p* < 0.05, with a *R*^2^ of 0.006, whereas significant negative relationship was shown between depression and being married, *r_pb_* = -0.073 (*N* = 726), *p* < 0.05, with a *R*^2^ of 0.005 (see **Table 6**). Compared to other marital statuses, divorcees were more depressed and married people were less depressed.

### Multiple Regression Analysis for Stress, HLOC, Age, Ethnicity, Occupation, and Marital Status in Predicting Depression

Multiple linear regression analyses were conducted to examine the effect of age, stress level, occupation (i.e., housewife, business owner, and professional), marital status (i.e., married, divorced) as well as internal- and powerful-others- HLOC on depression. The results showed all independent variables together significantly accounted for 12% of the variance in depression, *F*(10,685) = 9.355, *p* < 0.01 (see **Table 7**). However, when controlling for the interactions among variables, only stress, internal HLOC, Chinese ethnicity, and business owner and professional occupations remained significant predictors of depressive symptoms. Higher stress and working as a business owner predicted more depressive symptoms whereas higher internal HLOC, Chinese ethnicity, and professional occupations predicted lower levels of depressive symptoms.

**Table 7:** Summary of multiple regression analysis for age, stress, HLOC, occupation, and marital status in predicting depression.

Variable	*B*	*SE B*	β
Stress	1.482	0.306	0.178**
Internal HLOC	-2.014	0.473	-0.170**
Powerful-Others HLOC	-0.593	0.430	-0.055
Age	-0.063	0.039	-0.078
Chinese	-3.619	0.810	-0.167**
Housewife	-1.314	1.699	-0.031
Business Owner	4.170	1.565	0.103**
Professional	-5.603	1.639	-0.126**
Married	0.223	1.081	0.010
Divorced	2.981	3.078	0.036

*R^*2*^ = 0.120 (*p* < 0.01). *^∗^p* < 0.05*, ^∗∗^p* < 0.01*.

## Discussion

The aim of this study was to better understand the prevalence of depressive symptoms and their various correlates in Malaysia. We specifically looked at stress, HLOC, and a number of demographic factors such as gender, age, ethnicity, education, marital status, and occupation as potential predictors of depression among respondents from the states of Penang, Selangor, and Terengganu.

### Prevalence of Depressive Symptoms

Our data indicate an overall prevalence rate of 21% for moderate to severe depressive symptoms, which means that 1 out of 5 Malaysians in our sample were at-risk for depression. Specifically, looking at BDI-II scores, 10% of respondents reported having severe levels and 11% reported having moderate levels of depressive symptoms. These numbers are similar to the rates of severe depressive symptoms reported by some earlier studies using different measures on Malaysians. For example, previous studies reported rates of 11.3% (Wong and Lua, 2011), 12.3% (Tan and Yadav, 2015), and 10.3% (Kader Maideen et al., 2014). Similarly, if we combine rates for moderate, mild and severe symptoms from our study (about 35%), These numbers are more or less in line with the psychological distress estimate of 29.9% from the National Health and Morbidity Survey (Institute for Public Health, 2015). Based on this, we can say with some confidence that the prevalence of depression in Malaysia overall is significantly higher than that found in the United States and most other Western countries. The following sections examine some of the possible factors involved in this.

### Psychological Factors

#### Stress

Stress is experienced by everyone to varying degrees, but when experienced at high levels it is a powerful predictor of depression and other problems (Bergdahl and Bergdahl, 2002; Sangon, 2004; Rusli et al., 2008; Kader Maideen et al., 2014). As expected, Malaysians in our sample who reported greater stress tended toward more depressive symptomology. Controlling for other variables, stress was the strongest predictor of depressive symptoms among our participants. Future research should look more deeply into the major sources of stress among Malaysians with an eye toward preventive public policies.

#### Health Locus of Control

An interesting finding which contradicts those of many previous studies (Cheng et al., 2013; Aflakseir and Mohammad-Abadi, 2016) was that members of our sample who perceive their locus of control being centered on powerful-others reported less depressive symptoms. One possible explanation for this finding is the high level of power distance or hierarchical organization found in Malaysian society. Members of such societies generally expect care and patronage from social superiors and may take comfort in those expectations (Dysart-Gale, 2006). Similarly, Malaysia, in contrast to the United States and other countries where most locus of control research has been conducted is generally a collectivist culture, emphasizing interdependence among its members. In such cultures it is more normative to expect others to assume responsibility for one’s welfare (Hofstede, as cited in Dysart-Gale, 2006). On a related note, Malaysia is a very religious culture with about 98% of the population reporting membership in some religion (Bureau of Statistics, 2011). Such a high rate of religiosity may relate to a level of comfort with, and expectation of, one’s life being controlled by outside forces. On several levels, thus, we can see how the relationship of internal vs. external control and mental health may be culturally dependent.

On the other hand, our findings on the association between internal locus of control and depressive symptoms are congruent with previous studies (van Dijk et al., 2013). People with higher internal locus of control were also less likely to experience depressive symptoms (Vuger-Kovacic et al., 2007). For our sample, in other words, control was most important in minimizing depressive symptoms not so much whether it was internal or external. Interestingly, those who perceived high internal control also tended to perceive high external control. This pattern of perceived control should be explored further among Malaysians to see if it is indeed a unique aspect of their cultural worldview.

### Sociodemographic Factors

#### Ethnicity

Multiple linear regression analyses indicated that Malaysian Chinese ethnicity predicted lower levels of depressive symptoms compared to Malay and Indian ethnicities. Analyses showed that 26.7% of the “other” ethnicities, followed by 23.5% of Indians, 21.4% of Malays and 19.1% of Chinese had moderate to severe depression based on BDI-II. These findings are incongruent with Kader Maideen et al. (2014) study which found that ethnic Indians had the lowest prevalence of depressive symptoms, and with Fuad et al. (2015) who found no ethnic differences. On the other hand, our findings are similar to other studies that reported the highest prevalence of depressive symptoms among ethnic Indians, compared to Chinese and Malays (Yusoff et al., 2014; Kaur et al., 2014).

From a cross cultural viewpoint, it is noteworthy that Chinese ethnicity seems to be protective against depression. It is not known exactly how this mechanism works. However, previous research has found that Chinese tend to somaticize rather than psychologize their complaints when compared to other groups. Parker et al. (2001), for example, reported that Chinese were more likely to discuss somatic symptoms of depression (60% vs. 13%) compared to Australians. They also were less likely to report cognitive symptoms of depression, resulting in significantly lower total inventory scores.

#### Occupation

Professionals and housewives reported fewer depressive symptoms when compared to other occupations. Possibly this relates to professional occupations’ generally higher incomes, and greater autonomy, as well as access to support and other resources. Similarly, women who remain in the home likely come from middle- and upper-class backgrounds and experience better than average living standards with fewer major stressors. Housewives in this study were found to be significantly less stressed in comparison to other occupational groups. Interestingly, they were also significantly older (*M* = 47.74) on average than other groups. Likely not having young children, and not experiencing a need to generate income, these middle-aged housewives might be assumed to have better financial standing. Business owners, on the other hand, reported more depressive symptoms, possibly due to greater job pressure and longer working hours, which often relate to stress and poor health (e.g., Prottas and Thompson, 2006).

#### Other Demographic Factors

Consistent with most previous studies, divorcees reported more depressive symptoms compared to those who were married (Djernes, 2006; Blay et al., 2007; Taqui et al., 2007; Imran et al., 2009; Kader Maideen et al., 2014). Also, in accord with previous findings from Malaysia (Tan and Yadav, 2015) and the United States (American Psychiatric Association [APA], 2013), younger adults were more likely to report depressive symptoms. Our data, however, did not indicate that education level or gender relate to depressive symptoms. When controlling for other variables, stress level and business ownership were strong predictors of depressive symptoms; whereas higher internal HLOC, Chinese ethnicity, and professional occupations were protective.

### Application of Findings, Limitations and Future Studies

This study found a relatively high rate of moderate to severe depressive symptoms (21%) among Malaysian adults. Given that the participants in this study were mainly from urban areas, there is a possibility that urban-related stress may be contributing to this. Future studies should look at depression rates and associated factors in both urban and rural samples to clarify this issue. It is important to understand how modernization, work hours, traffic and congestion are influencing mental health in developing countries like Malaysia. It is quite possible that at least some of the dramatic increases in mental health challenges over the previous two decades indicated by the NHMS are related to changing environments and economic development. Policy makers need to be aware of the impact of urban and economic development on individual mental health and ensure that future development plans are undertaken with mental health concerns in mind. The influence of development-related factors such as rising housing and food costs, traffic congestion, crime, and reductions in green space on stress, depression and overall mental well-being should be explored as ways of addressing this issue from a public health perspective.

These data also suggest that Malaysians have a somewhat different perspective on locus of control from some other cultural groups. They seem to be able to trust authority figures for many of their needs related to well-being and not suffer psychologically for it. This could possibly be due to Malaysians being more collectivist in their mindset, expecting members of their social network to influence their thoughts, feelings, and behaviors (as cited in Cheng et al., 2013). Unlike western cultures where believing in powerful others or trusting in authority is seen as overly passive and relates to negative outcomes, Malaysians do not seem to have a conflict between possessing internal locus of control and trusting in others, and thus do not seem to experience negative repercussions from believing in powerful others. This finding warrants further exploration and, if supported, could be included in future intervention programs specifically tailored to Malaysians.

These data also indicate that professionals and housewives are protected against depressive symptoms whereas business owners are more vulnerable. However, we do not have any clear indication of the mechanisms underlying these differences. Future research should explore in more detail the interrelationships among occupation, stress, and well-being. In line with the government’s plan to transform Malaysia into a high-income nation by the year 2020, more attention needs to be paid to the aspects of rapid development that are conducive, or destructive, to mental well-being. The underlying factors related to Malaysian business owners’ depression, for example, seem particularly worth studying. Perhaps, for example, systems that support the needs of business owners (programs such as debt relief, tax credits, reduced cost health care, and child care services) could be deployed with the needs of this growing population, who contribute so much to our economy, in mind.

Other not-so-surprising findings related to marital status, with divorcees being at increased risk for depressive symptoms compared to married individuals. Future research might look at the challenges facing divorced individuals in Malaysia. Factors that could be explored include divorcees’ financial status and resources, number and age of children (if any), arrangement of work and childcare, leisure and entertainment opportunities, as well as psychological factors such as resilience/hardiness, self-esteem, and social support. Research might also explore the influence of social stigma against divorce that still exists among many groups in Malaysia.

This study had numerous limitations. First, these data were mostly collected from urban areas, meaning the participants might experience different stressors compared to those from more rural areas. Second, the ethnic composition of this sample varied somewhat from the population of Malaysia overall. Future research, thus, should include more data from suburban and rural areas, as well as a higher proportion of ethnic Malays. Also, because this study analyzed a preexisting data set, the selection of variables was limited. Ideally, future studies would include more of the many correlates of depression that have been identified in previous research. Future studies, for example, should look in more depth at such variables as social support, social skills and relationship quality, among others. Similarly, this study included only a simple measure of overall stress levels. It did not look at the more direct sources of Malaysians’ stress (e.g., financial issues, childcare, relationships, overwork, traffic, etc.). Additional studies should look at the specific stressors and life challenges facing Malaysians from various backgrounds. Furthermore, intrapersonal factors related to personal meaning, social identity and self-understanding can play important roles in mental health. Qualitative studies that examine the day-to-day experiences and meaning-making processes of modern Malaysians could be important to our understanding of their mental health issues. Overall, as Malaysia progresses on a path of rapid economic development and change, we need to be more aware of the many challenges that face its different peoples. Such knowledge should be a necessary part of how we shape future policies, and contribute to an overall aim of creating a mentally healthy, as well as prosperous, population.

## Author Contributions

SY was the primary author. GB and CT contributed data collection, design and analysis. CW was involved in the conceptualization and analysis of results.

## Conflict of Interest Statement

The authors declare that the research was conducted in the absence of any commercial or financial relationships that could be construed as a potential conflict of interest.
